# Celecoxib Decrease Seizures Susceptibility in a Rat Model of Inflammation by Inhibiting HMGB1 Translocation

**DOI:** 10.3390/ph14040380

**Published:** 2021-04-19

**Authors:** Hadeel Alsaegh, Hala Eweis, Fatemah Kamal, Aziza Alrafiah

**Affiliations:** 1 Department of Pharmacology, Faculty of Pharmacy, King Abdulaziz University, Jeddah 21589, Saudi Arabia; hadeel.z.alsaegh@outlook.com; 2 Department of Pharmacology, Faculty of Medicine, King Abdulaziz University, Jeddah 21589, Saudi Arabia; heweis@kau.edu.sa (H.E.); foakamel@kau.edu.sa (F.K.); 3 Department of Medical Laboratory Technology, Faculty of Applied Medical Sciences, King Abdulaziz University, Jeddah 21589, Saudi Arabia

**Keywords:** epilepsy, high-mobility group box protein 1, inflammation, oxidative stress, pilocarpine

## Abstract

The risk of developing epilepsy is strongly linked to peripheral inflammatory disorders in humans. High-mobility group box protein 1 (HMGB1) has the most focus for being a suspect in this scenario. The current study aimed to detect the celecoxib effect, an anti-inflammatory drug, on decreasing seizure susceptibility and organ damage in lipopolysaccharides (LPS)/pilocarpine (PILO) pretreated Wistar rats. Rats were divided into 6 groups (8 each): group 1 (control), group 2 (PILO), group 3 (PILO+LPS), group 4 (PILO+LPS+(VPA) Valproic acid), group 5 (PILO+LPS+Celecoxib), and group 6 (PILO+LPS+VPA+Celecoxib). LPS was used to induce sepsis and PILO to induce seizures. Oxidative stress markers, pro-inflammatory cytokines, and HMGB1 levels in serum and brain homogenate were evaluated. Histopathological studies were conducted on the hippocampus, liver, lung, and kidney. Treatment with celecoxib either alone or in combination with VPA significantly reduced Racine score and delays latency to generalized tonic-clonic seizures onset with a significant decrease in hippocampal levels of pro-inflammatory cytokines, oxidative stress markers, and increase in reduced glutathione. In addition, celecoxib treatment either alone or in combination with VPA suppressed HMGB1translocation into peripheral circulation more than treatment with VPA alone. Furthermore, hippocampus, liver, lung, and kidney histopathological changes were improved in contrast to other epileptic groups. Celecoxib either alone or combined with VPA has antiepileptic and multiorgan protective effects on acute seizures and inflammatory models induced by PILO with LPS. It decreased histopathological findings, oxidative, and inflammatory effects induced by VPA and LPS. This might be due to its anti-oxidative, anti-inflammatory and anti-HMGB1 mediated effects.

## 1. Introduction

Epileptogenesis is an involved modification in the typical brain structure that yields recurring seizures. Such a process is precipitated and aggravated by neuro-degeneration, disruption of the blood-brain barrier (BBB), amygdala, glutamatergic system, oxidative stress, and epigenetic modification of deoxyribonucleic acid (DNA). Since there is no efficient method yet, to modify or control this disorder’s pathway, novel therapeutic approaches are needed [1].

The risk of developing epilepsy, aggravating seizures frequency are strongly linked to peripheral inflammatory disorders in humans and animal studies, with the latter demonstrating an association between peripheral inflammatory bowel disorders and peripheral injection of Toll-like receptor 4 (TLR4) ligand lipopolysaccharide (LPS) and increased seizures frequency and their injuries [2]. Subtle injuries, chronic inflammation, gliosis, and brain nervous tissue microgliosis are crucial participants in epilepsy pathogenesis. Understanding the exact function and chemical mediators’ role and receptors involved in neuro-inflammatory reaction could help explain their contribution to epilepsy pathogenesis [3]. 

Epilepsy trials conducted on rodents detected a rapid, significant up-regulation of inflammatory markers expression in glia involved in epileptic activities. These inflammatory markers include interleukin (IL)-1β, IL-6, and tumor necrosis factor (TNF)-α, which are expressed in activated microglia and astrocytes and trigger a complement system, nuclear factor-kappa B (NF-κB), cyclooxygenase (COX)-2, chemokines and acute-phase proteins. These inflammatory reactions involve neuronal tissues and the endothelial layer of BBB. The fast release of high-mobility-group Box 1 (HMGB1) from microglia, neurons, and astrocytes after exposure to pro-convulsant insult and TLR signaling activation in astrocytes and neurons involved in triggering brain inflammation and reducing seizure threshold. Moreover, glial cell stimulation also triggers epilepsy, and this stimulation is mediated by HMGB1 through the TLR4/ NF-κB signaling pathway during seizures [4]. HMGB1 activates interleukin (IL)-1R/TLR signaling in nervous tissue. However, the anticonvulsant activity of TLR4 inhibitors and Box A., an endogenous HMGB1 competitor, indicates the involvement of glial cells and neurons secreted by HMGB1/TLR4 in the precipitation and continuation of seizures. Unfortunately, the detailed mechanism is not available yet [4].

Its role in epileptic pathogenesis has been the focus of many studies lately in which it has been reported to be involved in the disruption of the BBB and induction of cerebral inflammation. Studies conducted on rats have also strongly linked HMGB1 with the impaired cognitive abilities associated with neuroinflammation and epilepsy [4]. One of the latest managements for epilepsy that controls the generation and propagation of epileptic impulses following brain injuries is selective inhibition of the COX-2 enzyme. A restricted COX-2 ablation in forebrain neurons exerts neuroprotection, minimizing the brain inflammatory process that follows status epilepticus (SE) [5].

Celecoxib also was reported to be used as blockers for the COX-2 and HMGB1/TLR-4 pathways [6].

In this study the aim was to evaluate the effect of celecoxib on rat models of epilepsy induced by pilocarpine in rats pretreated with LPS for induction of inflammation.

The effect of celecoxib HMGB1 level was also investigated as a possible mechanism of celecoxib.

## 2. Results

### 2.1. Effects of Tested Drugs on Racine Score and Latency to Onset of Generalized Tonic-Clonic Seizure (GTCS)

**Figure 1:**
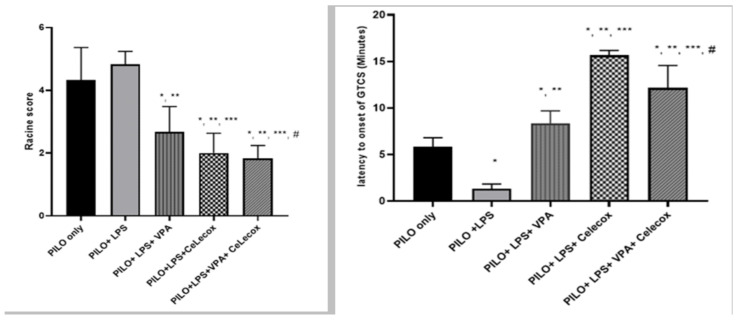
Effects of test drugs on Racine score and latency to Generalized Tonic-Clonic Seizure (GTCS) onset in rats subjected to Pilocarpine. Data were expressed as mean +/− standard deviation. PILO: pilocarpine, LPS: lipopolysaccharides; VPA: Valproic acid, Celecox: celecoxib. Comparison between groups was made using One-way ANOVA test followed by Tukey’s multiple comparison test. * *p* < 0.05 comparison versus PILO only; ** *p* < 0.05 comparison versus PILO+LPS, *** *p* < 0.05 comparison versus PILO +LPS+VPA, # *p* < 0.05 comparison versus PILO+LPS+Celecox.

A significant decline in Racine score and latency to GTCS onset was found in PILO+LPS+VPA and PILO+LPS+Celecoxib groups versus PILO PILO+LPS groups (*p* < 0.05). PI-LO+LPS+Celecoxib treatment showed a significant decrease in Racine score and latency to GTCS onset versus PILO+LPS+VPA (*p* < 0.05). There was decreased in Racine score and latency to GTCS onset in the PILO+LPS+VPA+Celecox group versus all other groups (*p* < 0.05) (Figure 1).

### 2.2. Oxidative Stress Markers

**Figure 2:**
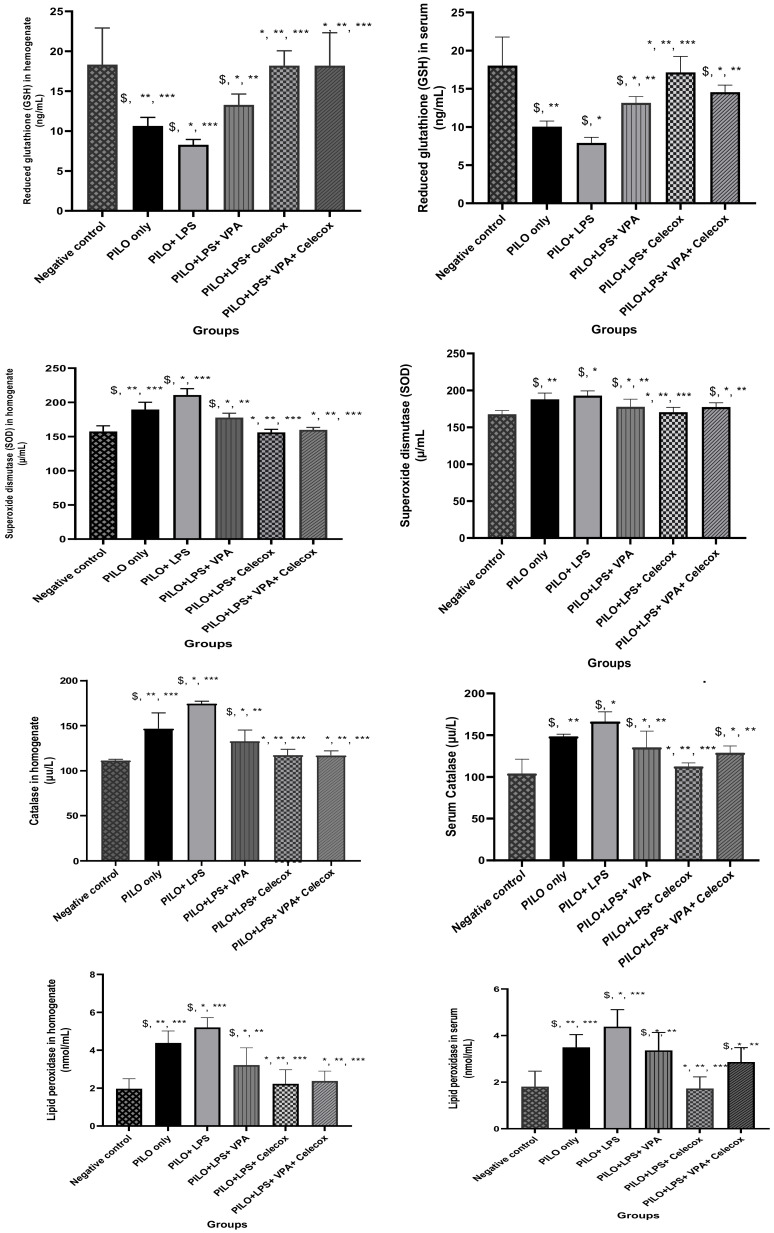
Effects of test drugs on oxidative stress markers in serum and hippocampus homogenate in rats of different groups. Data were expressed as mean +/− standard deviation. PILO: pilocarpine, LPS: lipopolysaccharides; VPA: valproic acid, Celecoxib: celecoxib. Comparison between groups was made using One-way ANOVA test followed by Tukey’s multiple comparison test. * *p* < 0.05 comparison versus PILO only; ** *p* < 0.05 comparison versus PILO+LPS, *** *p* < 0.05 comparison versus PILO+LPS+VPA, *p* < 0.05 comparison versus PILO+LPS+Celecoxib, $ *p* < 0.05 comparison versus negative control group.

The PILO and PILO+LPS group exhibited a significant reduction in glutathione (GSH) and superoxide dismutase (SOD) serum levels versus negative control rats (*p* < 0.05) and in the PILO+LPS group versus PILO (*p* < 0.05). GSH and SOD serum levels showed a significant increase in PILO+LPS+VPA, PILO+LPS+Celecox, and PILO+LPS+VPA+Celecox versus PILO and PILO+LPS groups (*p* < 0.05). Furthermore, GSH and SOD showed significant increase in PILO+LPS+Celecox versus the PILO+LPS+VPA group (*p* < 0.05). Catalase and lipid peroxidation serum levels exhibited a significant increase in PILO and PILO+LPS group versus negative control rats (*p* < 0.05) and in the PILO+LPS group versus PILO (*p* < 0.05). Catalase and LPO serum levels were significantly decreased in PILO+LPS+VPA, PILO+LPS+Celecox, and PILO+LPS+VPA+Celecox versus PILO and PILO+LPS groups (*p* < 0.05). Furthermore, there was a significant decrease in PILO+LPS+Celecox versus the PILO+LPS+VPA group (*p* < 0.05). In the PILO+LPS+VPA+Celecox group, serum levels of GSH, catalase, and LPO were significantly decreased while SOD was significantly increased versus PILO+LPS+Celecox (*p* < 0.05). The same changes were noticed in hippocampus homogenate levels (Figure 2).

### 2.3. Pro-Inflammatory Cytokines

**Figure 3:**
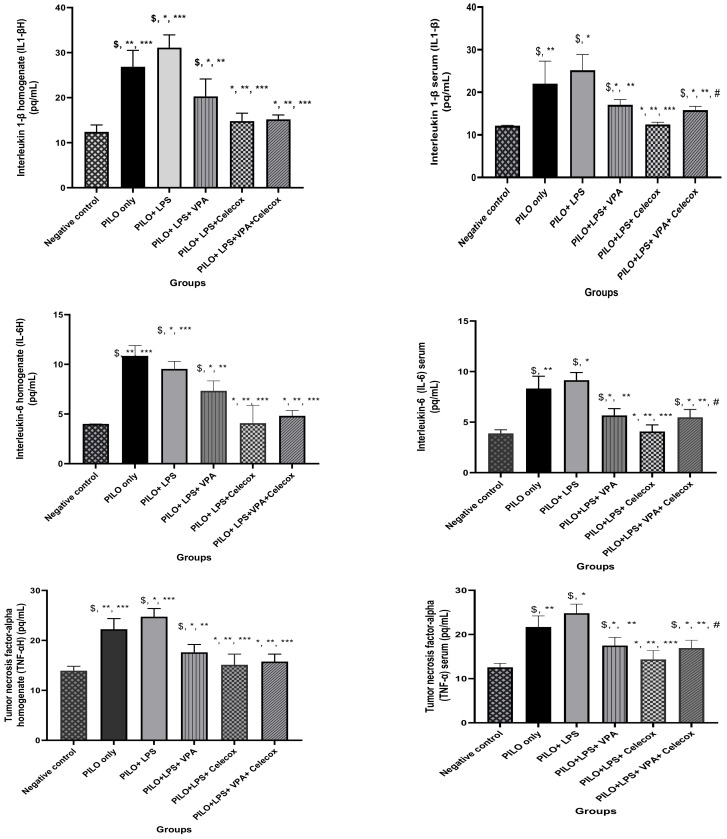
Effects of tested drugs on serum and homogenate levels of pro-inflammatory cytokines in rats of different groups. Data were expressed as mean +/− standard deviation. PILO: pilocarpine, LPS: lipopolysaccharides; VPA: valproic acid, Celecoxib: celecoxib. Comparison between groups was made using One-way ANOVA test followed by Tukey’s multiple comparison test. * *p* < 0.05 comparison versus PILO only; ** *p* < 0.05 comparison versus PILO+LPS, *** *p* < 0.05 comparison versus PILO+LPS+VPA, # *p* < 0.05 comparison versus PILO+LPS+Celecoxib, $ *p* < 0.05 comparison versus negative control group.

PILO and PILO+LPS group exhibited a significant increase in IL-1β, IL-6, and tumor necrosis factor alpha (TNF-α) serum and hippocampus homogenate levels versus negative control rats (*p* < 0.05) and in PILO+LPS group versus PILO (*p* < 0.05). IL-1β, IL-6, and TNF-α serum and hippocampus homogenate levels significantly decreased PILO+LPS+VPA, PILO+LPS+Celecox, and PILO+LPS+VPA+Celecox versus the PILO and PILO+LPS group (*p* < 0.05). Furthermore, there was a significant decrease in PILO+LPS+Celecox and PILO+LPS+VAP+Celecox versus PILO+LPS+VPA group in serum and homogenate (*p* < 0.05) and in PILO+LPS+Celecox versus PILO+LPS+VAP+Celecox group in serum (*p* < 0.05) (Figure 3).

### 2.4. Dynamic Changes of High Mobility Group Box-1 (HMGB1)

**Figure 4:**
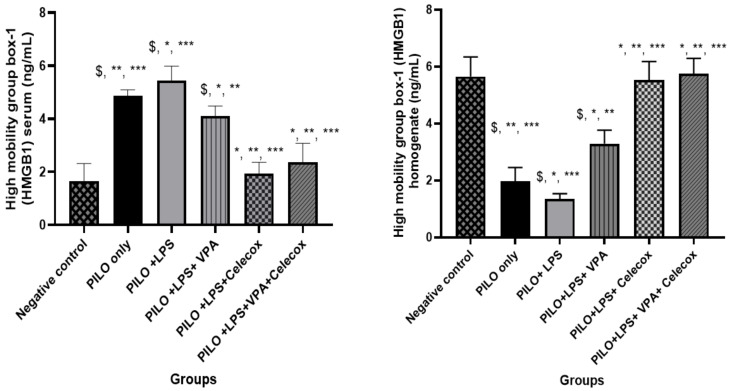
Effects of test drugs on high mobility group box-1 (HMGB1) levels in the serum and homogenate in rats of different groups. Data were expressed as mean +/− standard deviation. PILO: pilocarpine, LPS: lipopolysaccharides; VPA: valproic acid, Celecoxib: celecoxib. Comparison between groups was made using One-way ANOVA test followed by Tukey’s multiple comparison test. * *p* < 0.05 comparison versus PILO only; ** *p* < 0.05 comparison versus PILO+LPS, *** *p* < 0.05 comparison versus PILO+LPS+VPA, $ *p* < 0.05 comparison versus negative control group.

PILO, PILO+LPS, and PILO+LPS+VPA treated rats exhibited a significant decrease in HMGB1 brain homogenate (*p* < 0.05) versus negative control due to HMGB1 translocation. Treatment with VPA significantly inhibited HMGB1 translocation and significantly (*p* < 0.05) increased HMGB1 brain homogenate in PILO+LPS+VPA versus PILO and PILO+LPS rats. Interestingly, treatment with celecoxib only and combined celecoxib and VPA significantly inhibited HMGB1 translocation and increased the amount of HMGB1 brain homogenate in PILO+LPS+Celecox and PILO+LPS+VPA+Celecox groups versus PILO, PILO+LPS, and PILO+LPS+VPA treated rats (Figure 4).

### 2.5. Correlations between HMGB1 in Serum and Homogenate in Rats of Different Groups

**Figure 5:**
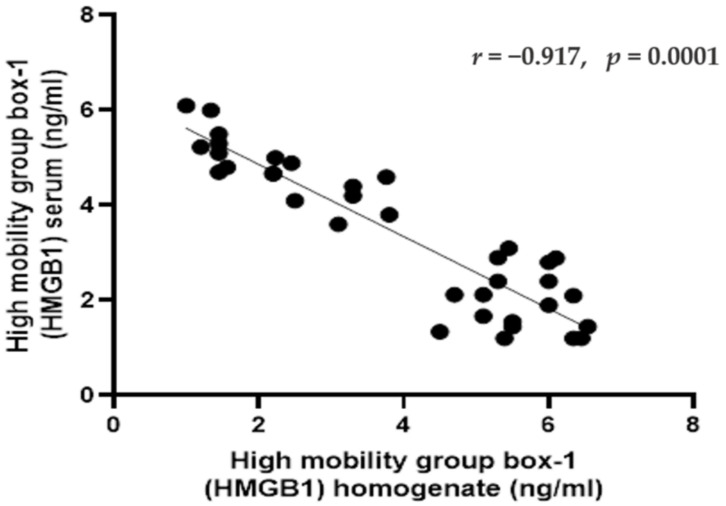
Pearson correlations between the high mobility group box-1 (HMGB1) (in the serum and homogenate) in rats in different groups. Data were expressed as correlation coefficient and significance. Correlation was made using Pearson correlation.

The serum level of HMGB1 exhibited significant negative correlation with HMGB1 homogenate level (*r* = −0.917, *p* < 0.001) (Figure 5).

### 2.6. Histological Results

#### 2.6.1. Hippocampus 

**Figure 6:**
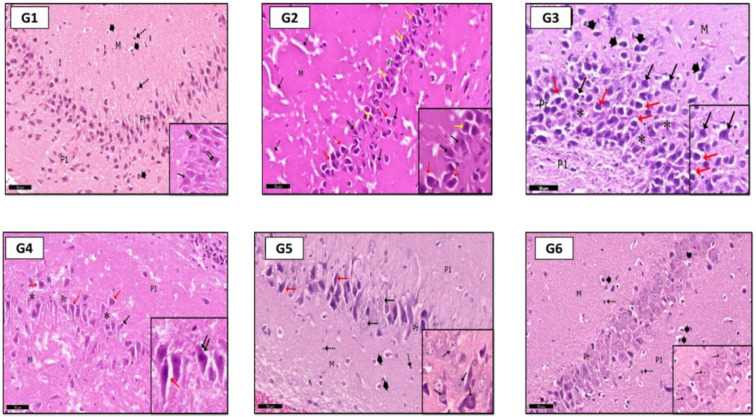
Photomicrographs of CA3 area of rat hippocampus shows: in control rats (**G1**), CA3 area consists of three layers; molecular (M), pyramidal (Pr), and polymorphic (P1) layers. The pyramidal cells are large neurons with rounded vesicular nuclei (↑), some of them are binucleated (inset—↑↑). Molecular layer (M) has a loose appearance with glial cells (head arrow) and interneurons (dot arrow). In (**G2**) (PILO rats), signs of degeneration were evident, especially in pyramidal layer in the form of shrunken pyramidal cells with pyknotic nuclei (yellow ↑) with wide perineuronal spaces (red ↑; some pyramidal cells have karyolitic nuclei (black ↑). Notice pale vacuolated areas (dot ↑) in molecular layer (M) and Polymorphic (P1) layer. In (**G3**) (PILO+LPS rats) higher degree of cellular degeneration was recorded with empty spaces (*), pyknotic nuclei (black ↑), shrunken neurons with perineuronal spaces (red ↑) within pyramidal layer (see also inset). Molecular layer (M) shows a marked enlargement of glial cells (head arrow). In (**G4**) (PILO+LPS+VPA rats), few shrunken pyramidal cells (black ↑) with perineuronal spaces (red ↑) within pyramidal layer (Pr); areas of pyramidal cells loss were localized (*). Inset: higher magnification of pyramidal cells. In (**G5**) (PILO+LPS+Celecoxib rats), moderate signs of improvement were recorded in most of pyramidal cells (black ↑); shrunken cells with perineuronal spaces (red ↑) were rare; glial cells (head arrow) and interneurons (dot ↑) in molecular (M) and polymorphic layers (P1). In (**G6**) (PILO+LPS+VPA+Celecoxib rats), cellular profile was nearly as in control rats, glial cells (head arrow), interneurons (dot ↑) and pyramidal cells appear with vesicular nuclei (↑). Insets are magnifications of pyramidal layer. Stain: H&E. Bar: 50 µm. Inset ×400.

In H&E stained sections of the hippocampus: In G2 (PILO rats), pyramidal cells of the CA3 region showed signs of degeneration; they were shrunken with acidophilic cytoplasm and pyknotic nuclei (Figure 6G2). G3 (PILO-LPS) revealed a marked decrease in normal pyramidal cells. Most of them were shrunken with pyknotic nuclei and dark cytoplasm (Figure 6G3); pale vacuolated areas were also seen in the deep molecular layer (Figure 6G3). G4 (PILO+LPS+VPA) revealed a mild decrease in normal pyramidal cells and a marked decrease in dark cells with pyknotic nuclei compared to groups 2 and 3. The molecular layer of CA3 showed marked pale vacuolated areas, enlarged interneurons, and enlarged glial cells (Figure 6G4). G5 (PILO+LPS+Celecox) rats showed that the CA3 region was histologically normal (Figure 6G5); however, occasional darkly stained cells were noticed in the pyramidal layer as compared to that of (PILO) and (PILO+LPS) groups. Examination of area CA3 of hippocampus proper of G6 (PILO+LPS+VPA+Celecox) rats showed marked improvement of their histological profile to be nearly normal (Figure 6G6).

**Figure 7:**
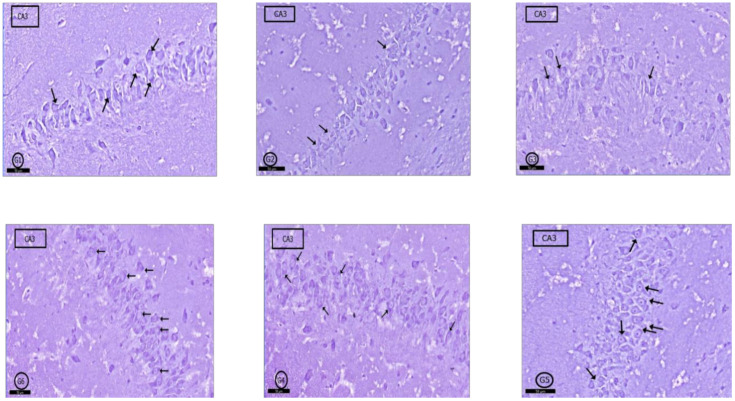
Photomicrographs of CA3 area of rat hippocampus proper: In control rats (**G1**), pyramidal cells showed heavily studded cytoplasm with Nissl granules (arrows). In (**G2**) (PILO rats) and (**G3**) (PILO+LPS rats), marked decrease in Nissl granules content was observed in pyramidal cells (arrows). On the other hand, Nissl granules were increased (arrows) mildly in (**G4**) (PILO+LPS+VPA rats), moderately in (**G5**) (PILO+LPS+Celecoxib rats) and markedly in (**G6**) (PILO+LPS+VPA+Celecoxib rats). Stain, toluidine blue. Bar 50 µm.

In toluidine blue-stained sections; G1 rats showed normal amounts of Nissl granules in pyramidal cells cytoplasm (Figure 7G1), but in G2 and G3, a marked decrease in Nissl granules was detected in the CA3 pyramidal cells (Figure 7G2,G3). In G4, a moderate increase in Nissl granules content was observed compared with that in G2 and G3 (Figure 7G4). Interestingly, more increase was found in Nissl granules amount in G5 and G6 than G4 in the pyramidal cells (Figure 7G5,G6).

#### 2.6.2. Liver

**Figure 8:**
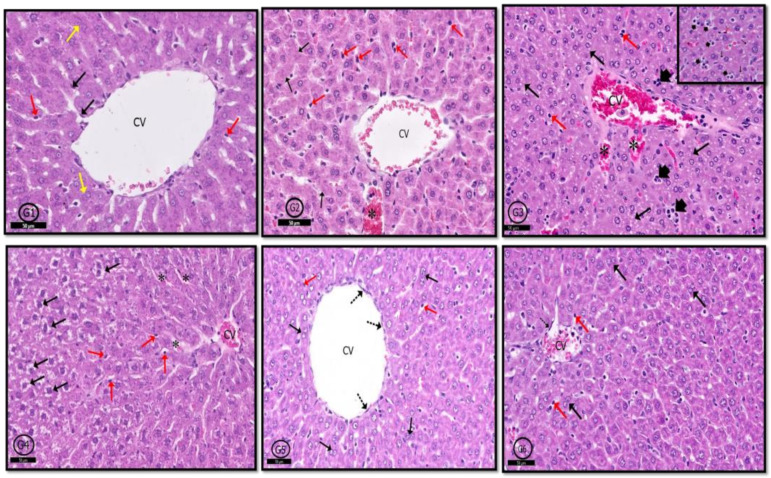
Photomicrographs of rat liver of different groups. In control rats (**G1**), normal hepatic profile contains hepatocytes arranged in cords around the central vein (CV), some hepatocytes are binucleated (black ↑); blood sinusoids (red ↑) and von Kupffer cells (yellow ↑). In (**G2**) (PILO rats), hepatic architecture was generally normal but few nuclei showed karyolysis (black ↑), congestion of some blood sinusoids (*), and few prominent von Kupffer cells (red ↑). In (**G3**) (PILO+LPS rats) and (**G4**) (PILO+LPS+VPA rats) hepatic tissue showed degenerated hepatocytes with pyknotic nuclei (black ↑), congested central vein (CV); focal aggregations of inflammatory cells (head arrow—inset) and increased von Kupffer cells (red ↑) in congested blood sinusoids (*). In (**G5**) (PLC+LPS+Celecoxib rats) and (**G6**) (PILO+LPS+VPA+Celecoxib rats), hepatic architecture was nearly normal showing polyhedral hepatocytes (black ↑), von Kupffer cells (red ↑) and newly formed hepatocytes (dot ↑). Stain; H&E. Bar 50 µm Inset ×400.

In G1 (control) rats, typical hepatic architecture was evident in the form of hepatic cords formed of hepatocytes and blood sinusoids (containing von Kupffer cells) arranged around central veins (Figure 8G1). In G2 rats, hepatic tissue was generally normal. However, mild congestion in blood sinusoids with hypertrophied von Kupffer cells in their walls was evident. Moreover, some hepatocytes showed karyolitic nuclei (Figure 8G2). Examination of G3 showed marked inflammatory cellular infiltration around central veins. Most of the hepatocytes around the central vein showed hydropic degeneration. Blood sinusoids and central veins were congested with hypertrophied intra-sinusoidal von Kupffer cells were seen (Figure 8G3). In G4 rats, signs of degeneration in hepatic tissue were clear; hepatocytes showed vacuolated cytoplasm, blood sinusoids and central veins were congested, and hypertrophied von Kupffer cells were localized between hepatocytes (Figure 8G4). Hepatic tissue in both G5 and G6 showed nearly normal profile picture as control rats; however, some specimens of G6 rats showed congested central veins and scattered inflammatory cells (Figure 8G5,G6). In this study, PILO induced continued seizure activity in contrast to the normal group. All rats reached stage five in the Racine score, as Jaworska-Adamu et al. [7] reported who used PILO with the same dose. Group 3 (PILO+LPS) gained continued seizure activity and decreased latency to GTCS onset versus the PILO group as recorded by Ho et al. [8]. The VPA only treatment group 4 (PI-LO+LPS+VPA) showed decreased Racine score and delay in latency to GTCS onset versus PILO and PILO+LPS groups. VPA reduced glutamate and aspartate release in LPS pre-condition pilocarpine-induced epilepsy rats [9] and increased synthesis and release of γ-aminobutyric acid (GABA) [10]. In the present study, the PI-LO+LPS+VPA+Celecox group showed significantly lower Racine score and delay in latency to GTCS onset versus PILO+LPS+Celecox group as others [11]. Exposure to COX-2 inhibitors, like celecoxib, stopped glutamate-mediated P-glycoprotein (P-gp) up-regulation and improved the efficacy of prescribed antiepileptic drugs [12]. 

#### 2.6.3. Renal Cortex 

**Figure 9:**
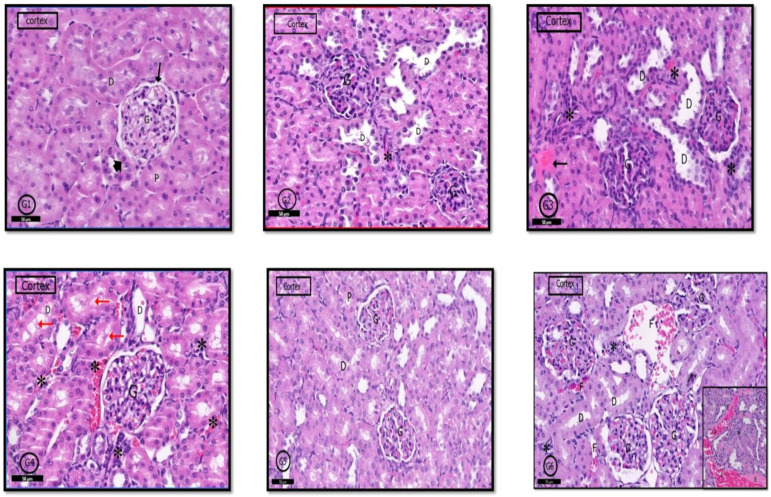
Photomicrographs of the rat renal cortex of different groups: In control rats (**G1**), normal renal cortical parts like glomerulus (G) in Bowman’s capsule (↑). (PCT) proximal convoluted tubules (D,P) and (DCT) distal convoluted tubules (head arrow) were seen. (**G2**) (PILO rats) showed localized areas of degeneration in the form of hypertrophied glomerular capillary loops (G) with dark nuclei. DCT had wide lumen (D); in addition to infiltrated inflammatory cells (*). In (**G3**) (PILO+LPS rats) and (**G4**) (PILO+LPS+VPA rats), signs of cortical degeneration were evident in the form of sclerotic glomeruli (G), wide DCT (D), infiltrated inflammatory cells with hemorrhage (*) and areas of exudate (↑). (**G5**) (PILO+LPS+Celecoxib rats) showed nearly normal renal cortical architecture of renal corpuscle (G), PCTs (P), and DCTs (D). In general, (**G6**) (PILO+LPS+VPA+Celecoxib rats) showed similar architecture as control rats but in localized areas (see also inset), collapsed glomeruli (C), interstitial infiltration (*) and focal interstitial widening with hemorrhage (F) and pink exudate (E) were seen. Stain: H&E. Bars: 50 µm Inset ×400.

H&E stained sections of the kidney from control rats (G1) showed normal renal cortex containing the renal corpuscles, proximal convoluted tubules (PCTs), and (DCTs) distal convoluted tubules Figure 9G1). The renal cortex in G2 rats was similar to that in control rats; however, few glomeruli have hypertrophied glomerular capillary loops with dark nuclei, and interstitial inflammatory cells infiltration was evident; lumens of some PCTs were dilated (Figure 9G2). In G3 and G4 rats, a wide range of degeneration signs was apparent in the renal cortex in the form of sclerosed glomeruli, pyknotic nuclei with an absence of glomerular space. Marked inflammatory cell infiltration and exudate were apparent in the cortical stroma. Many DCTs showed dilated lumens (Figure 9G3,G4). In G5 and G6 rats, marked improvement in the histological profile of the renal cortex was detected; however, some glomeruli in G6 rats showed obliterated capillaries, stromal inflammatory cells infiltration focal interstitial hemorrhages with exudate (Figure 9G5,G6). 

#### 2.6.4. Renal Medulla

**Figure 10:**
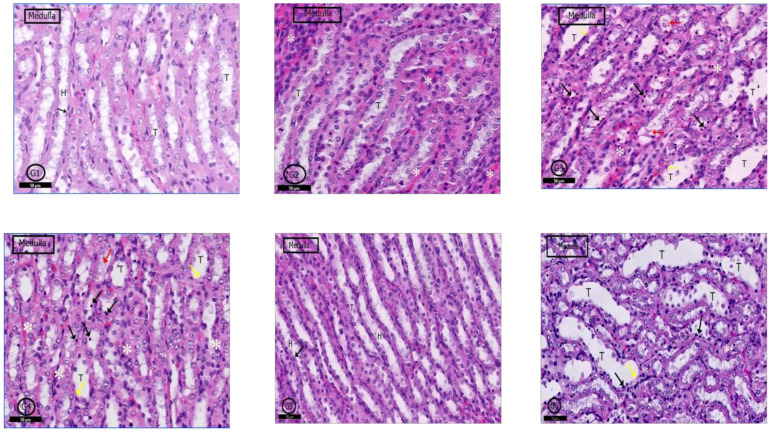
Photomicrographs of the rat renal medulla of different groups: In (**G1**) (control rats), the renal medulla was filled with normal collecting renal tubules (T) and loops of Henle (H). Loop of Henle lined by flattened cells with rounded nuclei (↑). In (**G2**) (PILO rats), renal medulla was normal but mild vascular congestion was observed (*). In (**G3**) (PILO+LPS rats) and (**G4**) (PILO+LPS+VPA rats) wide range of degenerated cells (black ↑), widen tubules (T) and congested blood vessels (*). Notice spread of the cellular casts (red ↑) and denuded lining (yellow ↑). (**G5**) (PILO+LPS+Celecoxib rats) showed nearly normal histological profile of renal medulla; the renal tubules (T) and loops of Henle (H) and round nuclei of their flattened cells (↑). (**G6**) (PILO+LPS+VPA+Celecoxib rats) showed degenerated cell lining (black ↑) and widened tubules (T). Notice denuded lining (yellow ↑) of the tubules and mild interstitial cell infiltrations with inflammatory cells. Stain: H&E. Bars: 50 µm.

A typical medullary profile was detected in (G1) control rats, collecting tubules with its cuboidal cells and loop of Henle with its flattened cells and round nuclei were clear (Figure 10G1). The renal medulla of G2 rats showed a nearly normal histological picture, but some tubular cells degenerated, and some blood vessels were congested (Figure 10G2). In G3 and G4 rats, the renal medulla showed dilated collecting tubules with degenerated cells, and cellular casts were clear; infiltrated inflammatory cells in the medullary stroma were also evident (Figure 10G3,G4). Examination of G5 and G6 showed nearly a similar picture to control rats (Figure 10G5); however, in G6, renal medulla in some areas showed dilatation of some tubules with scattered pyknotic nuclei and denuded lining; inflammatory cells infiltration was also detected (Figure 10G5,G6).

#### 2.6.5. Lung 

**Figure 11:**
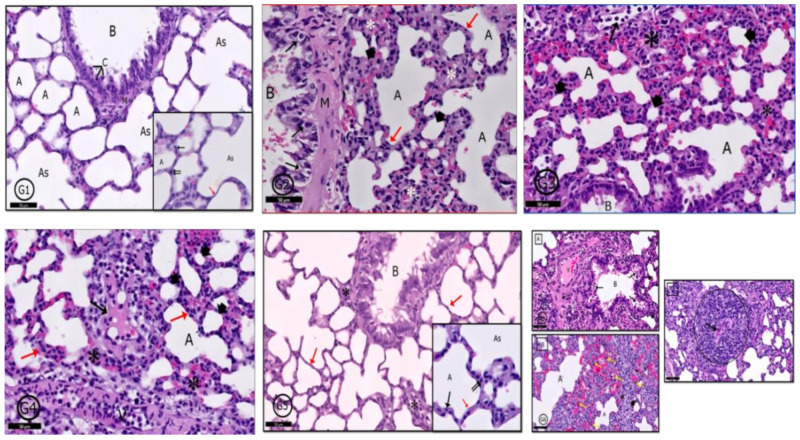
Photomicrographs of the rat lung of different groups: (**G1**) (control rats) showed normal pulmonary profile as a bronchiole (B), alveolar sacs (As), alveoli (A) separated by interalveolar septa (red ↑) and arterioles (Ar). Clara cells (C), pneumocyte type I (↑) and pneumocytes type II (↑↑) are normal bronchial and alveolar cells (see inset also). In (**G2**) (PILO rats), (**G3**) (PILO+LPS rats) and (**G4**) (PILO+LPS+VPA rats) a variety of degenerative and inflammatory cells were recorded; bronchiolar wall (**G2**-B) contains degenerated cells (↑). Mild thickening of alveolar walls in (**G2**) transformed into marked thickening in (**G3**,**G4**) with inflammatory cells infiltration in the three groups (*—red ↑ in **G2**,**G4**). Many alveoli showed emphysematous widening (A) and others showed narrowing of their lumens (head arrow). Some alveoli were occupied by inflammatory cells (↑—**G3**) and there was inflammatory exudate in the thickened interalveolar septa (↑—**G4**). (**G5**) (PLC+LPS+Celecoxib rats) showed nearly normal pulmonary tissues however, localized inflammatory cells infiltration (*) around a bronchiole (B). The interalveolar septa appear thin in most areas (red↑). Inset: Higher magnification of the pneumocytes shows pneumocyte type I (↑) and pneumocytes type II (↑↑). (**G6**): (PILO+LPS+VPA+Celecoxib rats) showed A: inflammatory cells infiltration (*) surrounding the bronchiole (B), degenerated cells in bronchiolar lumen (↑), congested blood vessels (V) and acidophilic exudate in the interstitium (dot ↑). B: Narrow alveolar spaces (head arrow) and others dilated (A) with marked inflammatory cells infiltration and extravasated RBCs (*). The marked black dot appears in the interstitial tissue showing activated macrophages (yellow ↑). C: Granuloma with central caseating materials (↑). Stain: H&E. Bar 50 µm. Inset ×400.

In control rats (G1), a typical pulmonary tissue profile was clear in the form of bronchioles, alveoli, alveolar sacs, and blood vessels. The alveolar epithelium was formed of; type I (flat) and type II pneumocytes (cuboidal) with rounded nuclei (Figure 11G1). In G2 rats, mild degenerative and inflammatory reactions were recorded in pulmonary tissues in the form of thickened interalveolar septa, widened emphysematous alveoli and extravasated red blood cells (RBCs) and inflammatory cells (Figure 11G2). In G3 and G4, a variety of degenerative changes were recorded in pulmonary tissues; some alveoli showed emphysema and others showed narrow lumens; interalveolar septa were markedly thickened; congested blood vessels; alveolar lumens and stroma contained inflammatory cells and extravasated RBCs beside stromal edema (Figure 11G3,G4). Examination of G5 showed marked improvement in tissue profile was recorded to be similar to that in control rats (Figure 11G5) but in G6, marked degenerative changes were clear as in G3 and G4, in addition to epithelioid granuloma, which appeared nearby bronchioles (Figure 11G6).

## 3. Discussion

The present study showed a significant decrease in GSH levels and a significant increase of LPO, SOD, and catalase levels in PILO and PILO+LPS rats’ hippocampus versus control levels. In addition, research showed that catalase activity, glutamate, and GSH were significantly increased following epilepsy, and ROS generation rise in CA1, CA3, and dentate gyrus with a marked caspase-3 expression [13]. In the current study, neurodegenerative changes were detected in pyramidal cells of the CA3 area of the hippocampus in epileptic model groups (PILO and PILO+LPS). These neurons appeared shrunken, deeply stained, and surrounded by perineuronal spaces. Feng et al. [14] stated that epilepsy leads to mitochondrial dysfunction in hippocampus rat neurons, resulting in apoptosis by triggering caspase three expressions. 

Mannaa and his colleagues [15] stated that valproic acid induces a significant MDA level reduction. Khamse et al. [16] mentioned that VPA significantly decreased oxidative stress than the pilocarpine-induced epilepsy group, but there was still a significant difference versus the control group. Interestingly, PI-LO+LPS+Celecox and PILO+LPS+VPA+Celecoxib group showed a significant reduction of lipoperoxidation, catalase, and SOD concentrations significant increase in GSH versus PILO and PILO+LPS groups as others [17].

In the present study, LPS administration resulted in a significant increase IL-1β, IL-6, and TNF–α versus negative control and PILO groups. Deng et al. [18] stated that stimulated astrocytes by LPS produced genes of IL-1β, IL-6, and TNF–α. Jaworska-Adamu and his colleagues [7] revealed the similarity of non-preconditioned and LPS preconditioned pilocarpine rodent epilepsy models in the pyramidal layer of CA1 and CA3 areas of the hippocampus proper on days 1 and 3. The current study showed that hematoxylin and eosin examination of CA3 area of hippocampus proper of PILO+LPS group exhibited marked glial cell enlargement in the molecular layer. Alyu and Dikmen [19] stated that IL-Iβ expressed in activated microglial cells and astrocytes improved glutamate release from astrocytes and reduced glutamate reuptake and thus, increases glutamate availability in neuronal synapses and promotes neuronal excitability. This could be explained by the significant increase in Racine score in group 2 (PILO) and group 3 (PILO+LPS). Furthermore, the present study showed a significant decrease in serum proinflammatory cytokines in group 4 (PILO+LPS+VPA) versus group 2 (PILO) and group 3 (PILO+LPS) as observed by Song et al. [20]. Wilson and his coworkers [21] stated that VPA diminishes ROS and proinflammatory cytokines, which are up-regulated in posttraumatic stress disorder. Previous studies [15] reported that hippocampus proper sections of pilocarpine-induced epileptic rats treated with VPA showed improved histological structure. Interestingly, the current study revealed that the celecoxib only treated group exhibited a significant decrease of proinflammatory cytokines (in serum) versus the VPA only group or VPA combined with celecoxib. However, celecoxib only showed a significant decrease of proinflammatory cytokines (in hippocampus homogenate) versus groups 2 (PILO), 3 (PILO+LPS), and 4 (PILO+LPS+VPA). Most pyramidal cells appeared with centrally located vesicular nuclei and cytoplasmic basophilia, as others [22]. Temp et al. [23] stated that PTZ increased cytokine levels in brain tissues and the hippocampus. Celecoxib and nimesulide attenuated proinflammatory cytokines in the cerebral cortex caused by PTZ. Fan et al. [24] reported that celecoxib treatment markedly attenuated chronic LPS-induced raises in active microglia and astrocytes, IL-1β and TNF-α concentrations, and phosphorylated protein levels p38 MAPK in neonatal rat brain. 

In the current study, an apparent decrease in Nissl granule content was detected in hippocampus neurons in epileptic models (PILO and PILO+LPS). Chromatolysis and reduced Nissl granules were found in Wistar rat neurons, mostly triggered by apoptosis [25]. Feng and his colleagues [14] reported that epilepsy in pilocarpine rat epilepsy models results in mitochondrial dysfunction in hippocampal rat neurons, leading to hippocampal neuron apoptosis. Celecoxib could increase the proliferation of neural precursor cells in the epileptic model and increased Nissl granules expression in hippocampus cells [26]. This might explain the increased content of Nissl granules in most pyramidal cells of area CA3 in the present study, which were detected by examining treated groups with celecoxib or VPA only or in combination versus epileptic model only or with LPS treated rats.

The present study results demonstrated that celecoxib treatment either alone or in combination with VPA suppressed HMGB1 extra localization outside the nucleus to extra-cellular space more than VPA alone treatment. HMGB1 translocation in neurons led to decreased HMGB1 levels in the brain and increased HMGB1 levels in the plasma of pilocarpine-induced epilepsy rats. These results support that HMGB1 was translocated and released from neuronal nuclei into surrounding areas, including the bloodstream. The release of HMGB1 into the CNS and peripheral bloodstream after pilocarpine injection led to the breakdown of BBB [27]. In the present study, there was a significant increase in IL-1β and HMGB1 in both pilocarpine only and pilocarpine with LPS. Fu and his colleagues [27] reported significant correlations between proinflammatory cytokine, IL-1β, and HMGB1. This study showed that celecoxib either alone or in combination with VPA significantly suppressed HMGB1 serum level versus the PILO+LPS+VPA group [6]. HMGB1 and TLR4 antagonists are used to increase the latency and reduces acute and chronic seizure recurrence [28].

Epilepsy patients may have renal or hepatic dysfunction that affects their antiepileptic treatment and seizures [29]. Jones and his colleagues [30] reported that 30 min after LPS injections, severe liver congestion, sinusoidal dilatation, edema, and hypertrophy of Kupffer cells were detected. VPA primarily metabolized in the liver by glucuronic acid conjugation, mitochondrial β-oxidation, and cyto-oxidation to produce multiple metabolites, some of which are biologically active and mediate hepatotoxicity triggered by VPA. As VPA induced up-regulation of inflammatory mediators as COX-2 [31], in the present study, the observed histological changes in the liver of rats following VPA might occur due to inflammatory and toxic effects of VPA. Liver cells exhibited degenerated swollen hepatocytes with clear cytoplasm surround congested central vein.

Moreover, multiple foci of macro-vesicular cytoplasmic vacuoles appeared with hypertrophied Kupffer cells as others [32]. In the current study, the liver of rats of celecoxib alone or VPA combined showed nearly a similar hepatic architecture as control as others [16]. VPA’s process causes liver damage depending partly on T-cells and macrophages’ activation to produce inflammatory cytokines [31]. Kupffer cells are primary sources of TNF-α production in response to bacterial LPS [33]. This could explain the presence of hypertrophied von Kupffer cells, which exhibited an apparent increase in the present study in PILO, PILO+LPS, and PILO+LPS+VPA groups.

The kidney and lung are the two most commonly affected organs in multi-organ failure syndrome. Renal insufficiency had an adverse influence on pulmonary function and acute kidney injury (AKI) [34]. In the present study, histological examination of both lung and kidney confirms a significant increase of proinflammatory cytokines in PILO, PILO+LPS, PILO+LPS+VPA PILO+LPS+VPA+Celecox treated groups versus the PILO+LPS+Celecox treated group. Furthermore, the PILO+LPS+VPA treated group exhibited marked interstitial infiltration and focal interstitial widening with hemorrhage and pink exudate during renal tissue histological examination. This group’s lung tissues showed marked mononuclear cellular infiltrations and epithelioid granuloma, which appeared nearby bronchioles as reported by de Abreu et al. [35] and Kim et al. [36]. Jones et al. [30] reported that bacterial LPS caused morphology changes in many tissues and triggers transformed lymphocytes, macrophage activation, and blood clotting. 

Moreoever, it stimulated oxidative stress and the release of pro-inflammatory cytokines from macrophages. All of this leads to multi-organ failure/dysfunction syndrome correlates with endotoxin shock outcomes. Xu and coworkers [37] reported that LPS-induced acute lung injury increased TNF-α production from the alveolar macrophage. Treatment with pilocarpine triggers TNF-α production from the alveolar macrophage. LPS-induced inflammatory responses were enhanced by pilocarpine. Valproic acid is known to induce several adverse effects involving hepatobiliary, renal, neurologic, hematological, cardiovascular, gastrointestinal, and metabolic systems. Rare has been found in the pulmonary system to have adverse effects of valproic acid, including acute eosinophilic effusion or diffuse alveolar hemorrhage. Kim and his colleagues [36] reported that valproic acid could induce lung injury such as drug-associated interstitial lung disease. Costalonga et al. [38] reported that VPA induces AKI. Mock and Schwetschenau [39] stated that valproic acid effects on kidneys contribute to hyperammonemia. Valproic acid causes more glutamine transported across the mitochondrial membrane in kidneys. Glutamine metabolized to glutamate, so more ammonia was produced and led to AKI. 

In the present study, the celecoxib only treated group showed nearly a similar histological picture as negative control rats in liver, lung, and kidney as others [16,40]. Roh and his colleagues [41] reported that celecoxib treatment attenuated smoking-induced changes in lung morphology, altered expression of various pro-inflammatory genes, and modulated activity of NF-κB signaling cascades. A previous study that examined celecoxib effects on hepatic ischemia/reperfusion (I/R) injury in rats reported that the celecoxib group showed well-preserved liver parenchyma with hepatocytes radially around the central vein; regular sinusoidal structures with normal morphology without any signs of congestion [42]. 

## 4. Materials and Methods

Fifty-eight adult male Wister rats aged 6–8 weeks, weighing 150–200 g, were used. The animals were delivered from the animal house of King Fahd Medical Research Center, Faculty of Medicine, King Abdulaziz University, Jeddah, Saudi Arabia. Animals were kept at a temperature of 23.2 °C, 12:12-h light/ dark cycle and free access to water and food, in groups of 8 animals per cage. A seven-day adaptation period was allowed before the beginning of the experiment. Animals were treated with good care, complying with ethical standards. Institutional approved the experimental protocol of Animals Care and Use Committee (ACUC) and Research Ethics Committee (REC) (reference #654-19).

### 4.1. Drugs and Chemicals

All chemicals were purchased from Sigma–Aldrich Co. (St. Louis, MO, USA). Pilocarpine HCL (PILO, catalog #6503), scopolamine methyl bromide (S.M.B., catalog #155-41-9), LPS from escherichia coli O55:B5 purified by phenol extraction (EC#297-473-0; Synonym: LPS; find Sigma-Aldrich-L2880 MSDS) and valproic acid (V.A, catalog # 1069-66-5) were supplied as white odorless powder dissolved in water. Celecoxib (catalog #169590-42-5) supplied as white odorless powder dissolved in dimethyl sulfoxide (DMSO). 

### 4.2. Methods

#### 4.2.1. Induction of Experimental Epilepsy 

According to the pilot study, all rats (*n* = 10) who received LPS in a dose of 10 mg/kg died, so the present study’s dose changed to 1 mg/kg. Rats received 0.25 mL of PBS via intraperitoneal injection (I.P.) route, and after 6 h, pilocarpine hydrochloride (PILO) was administered to induce seizure at a dose (380 mg/kg). To avoid muscarinic receptor activation, animals received S.M.B at a dose (1 mg/kg IP, 30 min before PILO injection) [7]. 

#### 4.2.2. Experimental Design

Rats were randomly allocated into six groups (*n* = eight each) as follows: Group 1 (negative control) rats received 0.9% normal saline by I.P. Group 2 (PILO–vehicle group) rats received 0.9% normal saline via I.P., and after 6 h S.M.B. 30 min before PILO (380 mg/kg I.P) injection to induce seizure [7,9,43]. Group 3 (PILO+LPS treated group) animals received (1 mg/kg I.P.) LPS then after 6 h, they received S.M.B. (1 mg/kg I.P) then after 30 min, they received PILO (380 mg/kg I.P) [9] (Sewal et al., 2017). Group 4 (PI-LO+LPS+VPA treated group) animals received V.A (250 mg/kg I.P.) then after 1 h LPS (1 mg/kg I.P.), after 6 h, they received a second dose of V.A (250 mg/kg I.P.) and S.M.B. (1 mg/kg I.P.) then after 30 min, they received PILO (380 mg/kg I.P.) [9]. Group 5 (PILO+LPS+Celecox) animals received celecoxib (10 mg/kg I.P.), then after 1 h, they received LPS (1 mg/kg I.P.). After 6 h, they received a second dose of celecoxib (10 mg/kg I.P.) and S.M.B. (1 mg/kg I.P.). After 30 min, they received PILO (380 mg/kg I.P.) [44]. Group 6 (PILO+LPS+VPA+Celecoxib) animals received celecoxib (10 mg/kg I.P.) after 1 h, they received LPS (1 mg/kg I.P.). After 6 h, they received a second dose of celecoxib (10 mg/kg I.P.) and V.A (250 mg/kg I.P.); after 30 min, they received S.M.B. (1 mg/kg I.P.), and after another 30 min, they received pilocarpine (380 mg/kg I.P.).

#### 4.2.3. Evaluation of Epilepsy

The animals were observed for 30 min after each PILO administration for convulsive behavior. Seizure activity was evaluated using the Racine scale [45]: Stage 0: no response. Stage 1: hyperactivity and vibrissae twitching. Stage 2: head nodding, head clonus, and myoclonic jerk. Stage 3: unilateral forelimb clonus. Stage 4: raring with bi-lateral forelimb clonus. Stage 5: GTCS with the absence of postural control. Latency to GTCS onset was recorded (minutes) at the end of 6 h. 

### 4.3. Biochemical Measurements

#### 4.3.1. Samples Collection

At the experimental end, rats were sacrificed by cervical dislocation after light ether anesthesia. Venous blood samples were collected from retro-orbital veins. Blood was placed into a plain tube and allowed to clot. Samples were centrifuged for 5 min at 3500 rpm, and supernatants were separated and stored at −20 °C until assayed. Animals were then decapitated in order to get brain, lung, kidney, and liver. Brain tissue was divided into two parts; the first was preserved at −80 °C for biochemical evaluation and the second for histopathological examination in 10% formaldehyde. Liver, kidney, and lung were maintained in 10% formaldehyde for histopathological examinations. 

#### 4.3.2. Brain Homogenate Preparation

The first portion of the brain was removed quickly from each rat and cleaned with chilled saline over ice. Hippocampus was quickly separated and immediately stored at –80 °C until biochemical assays. Tissue samples with an ice-cold phosphate buffer (pH 7.4) were homogenized at oscillation speeds (180–1800 oscillation in minutes) for hippocampus homogenate preparation using a TisssuLyser II homogenizer (Qiagen Cat. No./ID: 85300). The resulting suspension was subjected to two freeze-thaw cycles for further break cell membrane, and then the suspension was centrifuged for 15 min at 1500× *g*, then collected supernatant was stored at −80 °C till assayed.

#### 4.3.3. Biochemical Parameters in Hippocampal Homogenate and Serum

Enzyme-Linked Immunosorbent Assay (ELISA) kits were purchased from MyBiosource for rat’s GSH (catalog #MBS265966), rat’s LPO (lipid peroxidation) (catalog #MBS2515688), rat’s catalase (CAT, catalog #MBS726781), rat’s SOD (catalog #MBS036924), rat’s TNF-α (catalog #MBS355371), rat’s IL-1β (catalog #MBS825017), rat’s interleukin (IL)-6 (catalog #MBS726707) and rat’s high mobility group protein B1 (HMGB1) (catalog #MBS703437).

#### 4.3.4. Histopathological Examination

At the experimental end, the second half of the brain, liver, lung, and kidney were fixed in 10% neutral buffered formalin and processed to form paraffin blocks. Five μm thick sections had been serially cut from all blocks and subjected to hematoxylin and eosin (H&E) stain for routine histological examination. Toluidine blue staining was used to stain Nissl granules in neurons of hippocampus tissue. Sections were examined and photographed using a light microscope (model #BX51TF, Japan).

### 4.4. Data Analysis

Statistical analysis conducted using Statistical Package for Social Science Software package (SPSS) version 26. One-way analysis of variance (ANOVA) used followed by Tukey’s honestly as a post hoc test to compare study groups. Data presented as mean +/− standard deviation (SD). Significance set at <0.05 value. Graphs made by Graph pad prism software version 8 (USA).

## 5. Conclusions

Celecoxib, either alone or combined with VPA, had antiepileptic and multi-organ protective effects on acute seizures and inflammatory models induced by pilocarpine with LPS. Treatment with celecoxib either alone or in combination with VPA significantly reduced Racine score and delayed latency to generalized tonic-clonic seizures onset with a significant decrease in pro-inflammatory hippocampal levels cytokines, oxidative stress markers, and suppressed HMGB1 translocation into peripheral circulation more than VPA treatment alone. Furthermore, most hippocampus, liver, lung, and kidney histopathological changes were improved, which might be due to its anti-oxidative, anti-inflammatory and anti- HMGB1 mediated effects. Therefore, our data indicated that COX-2 inhibition would avoid epileptogenesis and possibly have significant therapeutic consequences in the future.

## Data Availability

Data are contained within the article.
